# Customized Intraoral Appliances for Self-Inflicted Oral Injuries in Pediatric Neurological Disorders: A Case Series

**DOI:** 10.7759/cureus.111164

**Published:** 2026-06-19

**Authors:** Alfaiciya Akber, Arun Mamachan Xavier, Balagopal Varma R, Suresh Kumar J, Parvathy Kumaran, Malini Venugopal, Nishna Thankappan

**Affiliations:** 1 Department of Pedodontics and Preventive Dentistry, Amrita Vishwa Vidyapeetham School of Dentistry, Kochi, IND

**Keywords:** dystonia, fires, glutaric aciduria type i, intraoral appliance, pediatric dentistry, post-neurosurgical bruxism, self-injurious behavior

## Abstract

Self-inflicted oral injuries in children with neurological disorders represent a significant clinical concern due to their multifactorial impact on oral and systemic health. Neurological disorders, such as febrile infection-related epilepsy syndrome (FIRES) and glutaric aciduria type I, may cause dystonia, involuntary orofacial movements, and self-inflicted biting, resulting in oral trauma. Management typically requires an interdisciplinary approach combining behavioral and pharmacologic strategies with protective intraoral appliances and, in selected cases, to prevent further trauma and preserve function. Three pediatric cases with neurological disorders in this series presenting with self-inflicted oral injuries were successfully managed with a customized intraoral appliance. All children showed mucosal healing within 7-10 days and no recurrence of trauma during the periodic follow-ups. Customized appliances provided effective protection, durability, and hygiene compatibility.

## Introduction

Neurological disorders in children often present with complex motor disturbances that significantly impact oral health and function. Among these, febrile infection-related epilepsy syndrome (FIRES) and glutaric aciduria type I (GA-I) are rare, but severe conditions are associated with movement abnormalities, dystonia, and recurrent seizures. FIRES is characterized by the sudden onset of refractory status epilepticus following a febrile illness in previously healthy children, frequently resulting in prolonged hospitalization and neurological sequelae [1,2]. GA-I, a metabolic disorder caused by a deficiency of glutaryl-CoA dehydrogenase, manifests with progressive dystonia and choreoathetoid movements leading to recurrent trauma to oral and perioral structures [3,4].

Orofacial manifestations in children with such neurological conditions include self-inflicted oral injuries, tongue and lip lacerations, bruxism, and soft-tissue ulcerations caused by involuntary movements or tonic spasms [5]. These injuries can cause pain, infection, bleeding, and functional limitations, such as difficulty in feeding, swallowing, and speech [6]. Moreover, repeated trauma delays healing and significantly affects the quality of life of both patients and caregivers [7].

Conventional management of self-inflicted oral trauma includes behavior modification, pharmacological control of dystonia, and protective intraoral devices [8]. However, many available appliances - such as mouthguards, soft splints, or bite blocks - are either bulky, poorly retained, or incompatible with oral hygiene maintenance, especially in pediatric patients with compromised neuromuscular control [9]. Therefore, an individualized approach that combines comfort, biocompatibility, and ease of use is essential.

Recent advances in digital dentistry and 3D printing have enabled the fabrication of customized intraoral appliances that are lightweight, precise, and easily reproducible [10]. These digitally designed devices offer improved fit, better patient tolerance, and simplified fabrication processes, making them suitable for medically complex pediatric patients [11]. They also allow integration of protective features, such as acrylic bite blocks or labial extensions, while maintaining minimal bulk and adequate ventilation [12].

Despite these advancements, current literature provides limited information regarding individualized intraoral appliance designs for preventing self-inflicted oral trauma in neurologically impaired pediatric patients, especially in intensive-care settings where conventional appliances are often poorly tolerated during dystonic or seizure-related movements.

This case series presents three pediatric patients with neurological disorders - FIRES, GA-I, and post-neurosurgical bruxism-managed successfully with customized intraoral appliances. The report highlights the role of digital technology in designing preventive devices that protect oral tissues, promote healing, and prevent recurrence of trauma.

This case series was prepared in accordance with the CARE (CAse REport) guidelines for clinical case reporting. Patient information, clinical findings, diagnostic assessment, therapeutic interventions, follow-up outcomes, and caregiver perspectives were documented systematically to improve transparency and reproducibility.

## Case presentation

This case series presents a patient-specific approach using customized appliances - including a digitally fabricated 3D-printed Neuro Bite Shield, modified Essix appliances, and ethylene vinyl acetate (EVA) soft splints - designed according to each child's neuromuscular pattern to improve protection, comfort, healing, and prevention of recurrent trauma.

To improve consistency and facilitate comparison of outcomes between cases, lesion severity and healing progression were evaluated using a structured clinical assessment protocol adapted from World Health Organization (WHO) criteria for traumatic oral lesions. The lesions were assessed based on their size, depth, presence of bleeding, edema, functional limitation, and recurrence.

Lesions were categorized as follows: Grade 1 (Mild): Superficial redness or mucosal indentation without ulceration or bleeding; Grade 2 (Moderate): Localized ulceration measuring less than 10 mm, associated with mild edema or occasional bleeding; and Grade 3 (Severe): Extensive ulceration greater than 10 mm with active bleeding, tissue tearing, marked edema, or functional difficulties affecting feeding, swallowing, or maintenance of oral hygiene.

Healing was evaluated during follow-up visits by assessing reduction in lesion size, degree of epithelialization, absence of bleeding, decrease in edema, and the absence of any new traumatic lesions.

Case 1

A seven-year-old female patient with FIRES was admitted to the pediatric intensive care unit following recurrent episodes of refractory seizures. According to the parents, the patient experienced three seizure episodes over a one-week period. The first episode occurred one week prior to presentation and required hospitalization at another institution. Following discharge, a second seizure episode occurred and subsided. However, a third and more severe seizure episode subsequently developed, leading to admission to our hospital. The patient demonstrated marked involuntary orofacial activity, including tonic mandibular contractions, dystonia, generalized movement disorders, and repetitive tongue thrusting. These manifestations resulted in multiple ulcerations involving the dorsal and lateral surfaces of the tongue. Parents additionally reported persistent bleeding from the affected areas.

**Figure 1:**
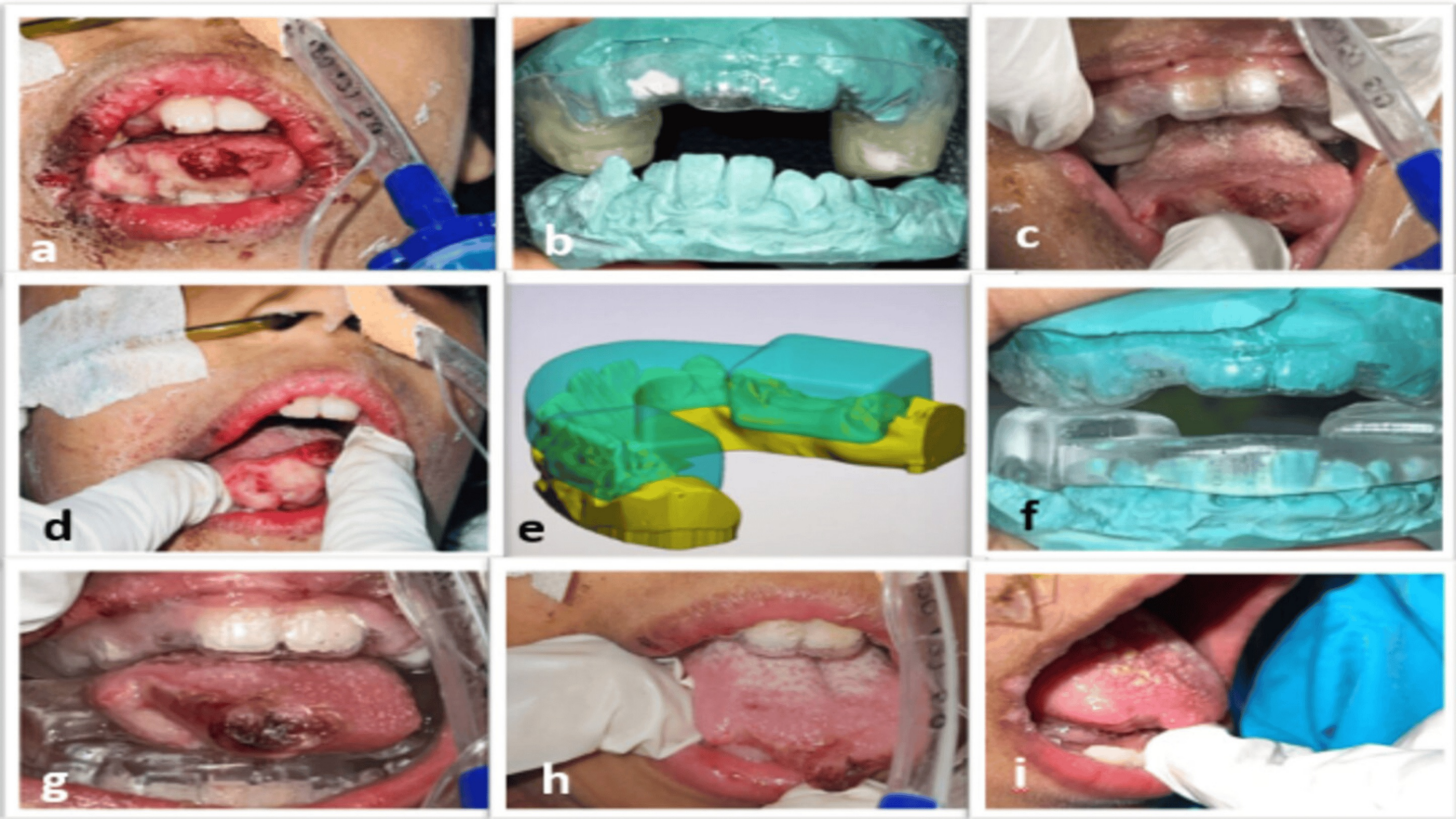
Clinical presentation of Case 1 (a) Pre-treatment intraoral view: the arrows point to the traumatic ulceration on the lateral surface of the tongue. (b) Modified Essix retainer with 10 mm bite block. (c) Intraoral view showing the modified Essix appliance positioned in the mouth. (d) Ulceration worsened due to inadequate trauma protection. (e) and (f) Digitally designed modified Essix retainer with posterior acrylic bite block and 3D-printed Neuro Bite Shield. (g) The arrow denotes scab formation on the lateral border of the tongue. (h) Post-treatment intraoral view: the arrow denotes significant mucosal healing 10 days following appliance delivery. (i) The arrow denotes complete mucosal healing at two-month follow-up.

On extraoral examination, no facial asymmetry was observed. Intraoral evaluation revealed a 10 × 8 mm traumatic ulcer on the right lateral border of the tongue, along with diffuse erythema, mucosal tearing, and indentations corresponding to dental impressions (Figure 1a). The patient exhibited pronounced dystonic movements with contortion of the facial musculature. Mandibular activity was continuous and uncontrolled, repeatedly forcing the tongue between the teeth, resulting in ulcerations of varying dimensions, including a laceration on the anterior one-third of the tongue. Based on the WHO criteria, the lesion was categorized as Grade 3 (Severe) due to extensive ulceration, recurrent bleeding, mucosal tearing, and functional compromise associated with continuous involuntary mandibular activity. Although the patient was sedated, uncontrollable self-biting episodes recurred whenever consciousness returned. Due to the altered sensorium and persistent seizure activity, the patient was unable to respond to commands, necessitating a passive and atraumatic approach to oral management.

An immediate maxillary and mandibular putty impression was obtained while the child was under controlled sedation. Casts were poured, and a vacuum-formed thermoplastic Essix retainer modified with a 10-mm acrylic bite block was fabricated and delivered (Figures 1b, 1c). This appliance increased the interocclusal space to prevent further tongue entrapment during dystonic movements. However, the device demonstrated several limitations, including susceptibility to fracture, reduced trauma-protective capacity, and poor retention stability, leading to progression of the ulcerative lesion (Figure 1d).

Due to these drawbacks, a digital workflow was initiated the following day. The dental cast was scanned using an intraoral scanner (CEREC Omnicam), and the digital model was finalized and exported as a Standard Tessellation Language (STL) file. The digital restorative process followed three standard steps: first, the dental arches were scanned to capture precise geometric data; next, the scanned information was digitally manipulated using computer-aided design (CAD) software to design the appliance and incorporate the required bite block dimensions; and finally, the completed design was used for additive manufacturing of the final appliance (Figure 1e).

Using this workflow, a custom 3D-printed Neuro Bite Shield was fabricated, incorporating a 7-mm bite block. The appliance was produced using Dental LT Clear (DLC), a biocompatible acrylic light-polymerizing clear resin (Formlabs, Somerville, MA). Printing was performed using the Formlabs 3BL printer, with a 0.1 mm slice thickness. The initial Essix acrylic block was selectively reduced to 3 mm, and with the addition of the 7-mm Neuro Bite Shield, a total of 10 mm interocclusal opening was achieved (Figure 1f).

The 3D-printed appliance was finished with smooth, soft-tissue-friendly borders and delivered at bedside, ensuring passive retention and absence of overextension (Figure 1g). Parents were instructed to maintain the device with periodic chlorhexidine cleansing and to continue routine oral hygiene using moist gauze swabbing.

Active healing of the tongue was observed within the initial days following appliance insertion. By day seven, the ulcer demonstrated substantial epithelialization, and complete healing was noted by day 10. No new traumatic lesions developed during this period (Figure 1h).

The appliance remained stable throughout involuntary movements and seizure episodes, showing no episodes of dislodgment or structural compromise. It was well tolerated, with no associated mucosal irritation or discomfort. Parents reported improved comfort, reduced anxiety regarding recurrent bleeding episodes, and easier maintenance of oral hygiene following appliance therapy.

At the one-month follow-up, the patient continued to remain free of recurrent oral trauma. A two-month review confirmed complete healing and maintained protection, indicating sustained effectiveness of the customized appliance in preventing further self-inflicted injury (Figure 1i).

Case 2

A three-year-old male diagnosed with GA-I, accompanied by infantile dystonia, was referred from pediatric neurology for management of recurrent self-inflicted lip and buccal mucosal injuries. The child exhibited prominent dystonic and choreoathetoid movements, which intensified during stress and feeding episodes. According to the parents, frequent self-biting of the lower lip resulted in persistent swelling, ulcerations, scarring, and significant difficulty in maintaining adequate oral hygiene.

**Figure 2:**
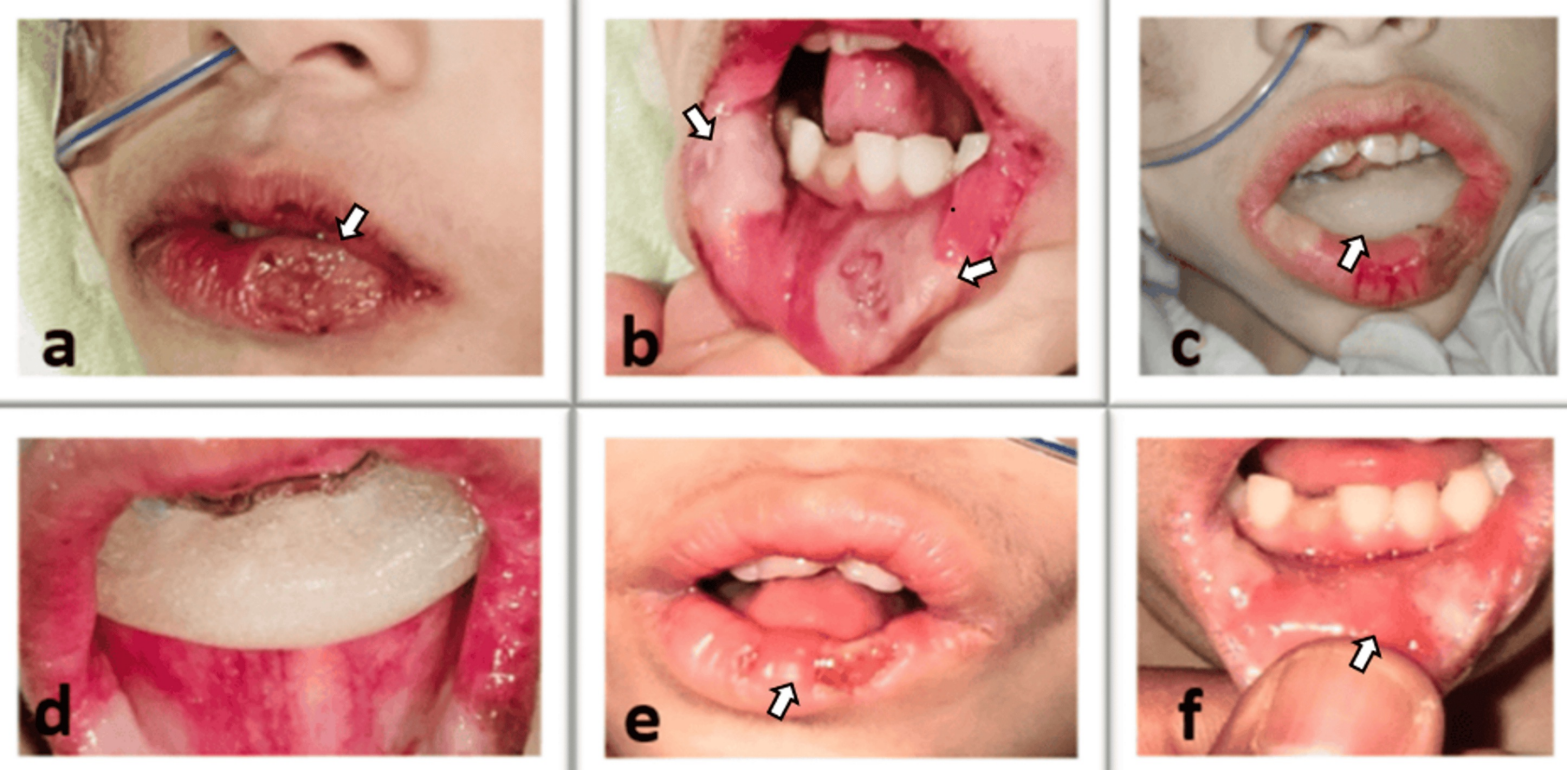
Clinical presentation of Case 2 (a) and (b) Pre-treatment intraoral view: the arrows indicate lower lip swelling and ulceration. (c) The arrow points to the modified Essix appliance with custom labial acrylic extension. (d) Intraoral view of the modified Essix appliance. (e) and (f) The arrows indicate healing of the lower lip mucosa on day eight following appliance placement.

Extraoral and intraoral examination revealed hypertrophy of the lower lip with multiple areas of crusted ulceration. Frictional keratosis was evident along the labial mucosa, and traumatic ulcerations were present on both the inner and outer surfaces of the lower lip (Figures 2a, 2b). The child was in the primary dentition stage, and no sharp incisal edges were identified that could have contributed to the injury. Continuous repetitive dystonic jaw movements were observed during clinical evaluation, corroborating the history of involuntary self-biting. According to the WHO criteria, the injury was graded as Grade 2 (Moderate), as the patient presented with localized ulcerations, edema, crusting, and recurrent soft-tissue trauma without active uncontrolled bleeding or deep tissue laceration. Clinical assessment included evaluation of lip swelling, ulcer dimensions, feeding difficulty, and recurrence of self-biting episodes.

Maxillary and mandibular putty impressions were obtained under controlled sedation using Pedicloryl syrup (25 mg/kg), and the corresponding casts were poured. A vacuum-formed thermoplastic Essix appliance was fabricated, chosen for its lightweight design, comfort, and minimal bulk - characteristics well-suited for a child experiencing involuntary orofacial movements.

The appliance design included a full-coverage Essix shell for the mandibular arch, complemented by a custom labial acrylic extension projecting anteriorly to shield the lower lip from the incisors. Additionally, smooth, rounded contours were incorporated throughout the appliance to prevent soft-tissue irritation during dystonic episodes (Figures 2c, 2d).

Parents received detailed instructions regarding appliance insertion and removal, daily hygiene protocols, and monitoring for signs of wear, distortion, or debris accumulation.

Marked improvement in the ulcerated labial mucosa was noted within five days of appliance use. Complete healing was achieved by the third week, accompanied by a significant reduction in edema and local inflammation. The child tolerated the appliance well during feeding and routine activities, and no dislodgment occurred throughout use.

During the six-week follow-up period, although dystonia persisted, the child remained free of new self-inflicted injuries, with a progressive reduction in lip swelling (Figures 2e, 2f). Parents reported improved comfort, easier oral hygiene maintenance, and better feeding behavior, reflecting favorable functional and protective outcomes.

Case 3

A two-year-old male admitted to the pediatric intensive care unit (PICU) under pediatric neurology was referred for evaluation of persistent teeth grinding associated with bleeding from the lower incisor region. The child had undergone neurosurgical intervention involving laminectomy and excision of an extramedullary intracranial mass lesion four days prior and was currently recovering with associated diaphragmatic palsy.

**Figure 3:**
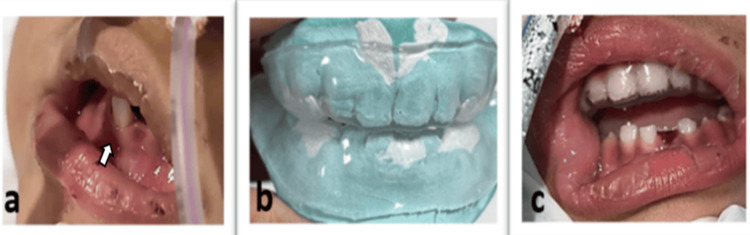
Clinical presentation of Case 3 (a) The arrow indicates labial mucosal trauma. (b) Bilateral soft splints delivered. (c) Post-treatment intraoral image showing resolution of bruxism on day 10.

Parents reported episodes of nocturnal bruxism and rhythmic masticatory muscle activity, resulting in repeated grinding and soft-tissue trauma to the lingual mucosa. Intraoral examination revealed Grade 3 mobility of tooth 71 (Fédération Dentaire Internationale system) with associated gingival bleeding, occlusal wear facets on the mandibular molars, and ongoing involuntary biting despite sedation (Figure 3a). Based on the WHO assessment protocol, the lesion severity was classified as Grade 2 (Moderate) due to gingival bleeding, traumatic mucosal injury, and parafunctional wear associated with persistent bruxism, without extensive tissue loss or deep ulceration. Bruxism intensity, mucosal healing, and recurrence of traumatic injury were monitored during follow-up visits. With parental consent, tooth 71 was extracted under topical local anesthesia due to its severe mobility and the risk of further soft-tissue injury. Considering the child's reduced neuromuscular control and persistent parafunctional activity, bilateral soft splints were planned to provide occlusal cushioning, minimize discomfort, and protect the oral tissues.

Maxillary and mandibular impressions were recorded, the casts were poured, and all undercuts were appropriately blocked out. Soft splints were then fabricated from 2 mm medical-grade EVA for both arches (Figure 3b). The maxillary soft splint was well-adapted and stable and showed good intraoral retention, whereas the mandibular soft splint was repeatedly displaced by the patient through tongue movements, limiting its long-term retention.

Despite this limitation, the maxillary splint offered effective protection. A noticeable reduction in the intensity of bruxism episodes was observed following appliance placement, and no further soft-tissue injuries occurred.

Parents were educated on nightly appliance use, cleaning techniques using chlorhexidine-soaked gauze, and the importance of maintaining adequate oral hygiene.

By day 10, bruxism episodes showed decreased intensity, and no new cheek or mucosal trauma was noted (Figure 3c). At the one-month and three-month follow-up visits, the maxillary soft splint remained intact, well-tolerated, and easy for parents to maintain. The appliance continued to provide consistent soft-tissue protection despite ongoing diaphragmatic palsy and intermittent involuntary movements.

Written informed consent was obtained from the parents/guardians of all patients for publication of the clinical findings, treatment details, and accompanying clinical photographs included in this case series. All efforts were made to maintain patient confidentiality and anonymity.

Table 1 presents the summarized version of the three pediatric neurological cases managed with customized intraoral appliances.

**Table 1:** Summary of the three pediatric neurological cases managed with customized intraoral appliances EVA: Ethylene vinyl acetate. Table credit: Dr. Alfaiciya Akber

Parameter	Case 1	Case 2	Case 3
Age/Sex	7-year-old female	3-year-old male	2-year-old male
Neurological Condition	Febrile infection-related epilepsy syndrome (FIRES)	Glutaric aciduria type I	Post-operative neurosurgical bruxism
Primary Oral Injury	Lateral tongue ulcer due to involuntary biting	Lower-lip ulceration from dystonic movements	Bilateral teeth grinding and posterior teeth wear
Clinical Features	Involuntary jaw contractions, tongue thrusting, altered sensorium	Dystonia, choreoathetoid movements, lip hypertrophy	Rhythmic masticatory muscle activity, nocturnal bruxism
Appliance Fabricated	Modified Essix retainer + acrylic bite block + 3D-printed neurobite shield	Modified Essix appliance with labial acrylic extension	Bilateral soft splints
Fabrication Method	Digital extratraoral scan + 3D printing	Vaccum formed appliance	Vaccum formed soft EVA splint
Key Features	Tongue protection, occlusal separation, lightweight design	Lip protection, minimal bulk, high adaptability	Cushioning during grinding
Healing Time	9 days	8 days	10 days
Tolerance and Retention	Excellent, stable during seizures	Good, requires caregiver assistance	Excellent, well-tolerated at night
Follow-Up Outcome	No recurrence at 1- and 3-month review	No new injuries over 6 weeks	No cheek trauma at 1- and 3-month review

## Discussion

Self-injurious oral trauma is a well-recognized complication in patients with neurological and movement disorders, arising from impaired sensory feedback, involuntary motor activity, cognitive deficits, or behavioral dysregulation. Such trauma may include repetitive biting of the lips, tongue, buccal mucosa, or perioral tissues, often leading to ulceration, infection, scarring, and functional impairment [13,14]. Early identification is essential, as chronic injuries can worsen nutritional status, interfere with speech, and significantly affect quality of life. Management typically involves a multidisciplinary approach, using behavioral strategies, pharmacologic modulation of movement disorders, and customized intraoral appliances designed to redistribute forces and protect vulnerable tissues [15].

When providing customized intraoral appliances for patients with neurological disorders, key considerations include the type and severity of the disorder, frequency of involuntary movements or seizures, age, dentition stage, parafunctional habits, medical status, and caregiver support. The appliance should offer effective protection, good retention, smooth contours, soft or flexible materials, and a safe design - especially for patients prone to seizures. Dental factors, such as occlusion, tooth alignment, and oral health, influence the fit and stability. Tolerance, minimal interference with speech or swallowing, and clear instructions on wearing duration are also important. Regular follow-up, proper hygiene, and timely replacement of worn or loose appliances ensure continued safety and effectiveness [13].

An important strength and distinctive aspect of the present case series was the individualized appliance design tailored to the specific neurological condition, pattern of self-inflicted oral injury, neuromuscular status, age, and tolerance level of each child. Rather than relying on conventional prefabricated mouthguards, the appliances were modified according to the functional requirements and behavioral challenges of each patient. This patient-centered approach allowed selection of different materials and designs depending on the severity of involuntary mandibular movements, site of trauma, and anticipated compliance.

Although previously reported acrylic-based appliances have shown favorable healing outcomes, several limitations remain. The digitally fabricated acrylic bite plate described by Garcia et al. incorporated full palatal acrylic coverage, stainless steel wire components, and rigid acrylic shielding, which may increase appliance bulk, reduce patient comfort, and predispose to soft tissue irritation in children with involuntary mandibular movements [16]. The need to remove the initial labial shield because of interference with involuntary movements further highlighted challenges related to appliance tolerance and functional adaptation [16]. Case 1 demonstrated that, while conventional Essix appliances may provide temporary protection, they may not withstand intense dystonic forces. Transitioning to a digitally designed and 3D-printed Neuro Bite Shield improved structural durability, retention, and patient comfort. Recent advances in digital dentistry, including CAD-based design and additive manufacturing, permit fabrication of highly precise and patient-specific appliances with improved reproducibility, material strength, and adaptability [10,11]. The successful use of Dental LT resin in this case is consistent with emerging evidence supporting digitally fabricated protective appliances for neurologically impaired pediatric patients [12].

Similarly, the noninvasive intraoral acrylic appliance described by Waseem et al. utilized a bulky mandibular acrylic splint with increased vertical dimension, which may adversely affect speech, feeding, oral function, and long-term adaptability [17]. The rigid auto-polymerizing acrylic structure also predisposes to mucosal irritation, pressure areas, fracture, and repeated adjustments during growth and dentition changes [17]. In addition, conventional fabrication procedures requiring physical impressions and acrylic processing may be difficult in neurologically compromised or uncooperative pediatric patients and may occasionally necessitate sedation. In comparison, Essix-based appliances with customized labial acrylic extensions provide a more conservative and patient-friendly alternative because of their reduced bulk, smoother margins, improved flexibility, enhanced esthetics, and greater patient comfort [8,9]. Case 2 highlighted these advantages, as the modified Essix appliance effectively prevented lip entrapment without compromising feeding or comfort and was better tolerated than rigid protective appliances.

A rigid acrylic appliance reported by Galeotti et al. effectively prevented recurrence of self-inflicted trauma [9]. However, hard acrylic appliances generally provide limited shock absorption during involuntary jaw movements and may contribute to discomfort, appliance fracture, soft tissue irritation, and reduced patient compliance [9]. In contrast, soft medical-grade EVA splints offer greater flexibility, improved force distribution, enhanced cushioning, and reduced risk of secondary trauma during seizure-related mandibular spasms. Furthermore, EVA-based appliances may better accommodate erupting dentition and dynamic oral movements in pediatric patients, making them a safer and more adaptable conservative option for neurological seizure disorders. Case 3 demonstrated the clinical utility of soft EVA splints in reducing bruxism intensity and eliminating soft-tissue injury following neurosurgical recovery. Although mandibular retention was inconsistent, the maxillary EVA splint alone provided effective occlusal cushioning and significantly reduced trauma, supporting the reported benefits of soft splints in the management of pediatric parafunctional habits and self-inflicted oral injuries.

The use of WHO standardized lesion severity grading and follow-up assessment criteria in the present series improved clinical documentation, facilitated objective evaluation of healing outcomes, and may support comparison with future studies involving self-inflicted oral injuries in neurologically compromised pediatric patients. Early dental intervention is vital for preventing worsening trauma, secondary infection, and feeding difficulties in neurologically compromised children (as presented in Table 1). In this series, timely use of protective appliances led to faster healing, fewer self-inflicted injuries, better feeding and hygiene, and reduced caregiver stress, consistent with previous findings [5-7]. Clinically, the cases highlight the need for individualized appliance design, the advantages of digital workflows for rapid and precise fabrication in critical-care settings [10-12], and the benefits of soft or low-profile materials for younger or highly dystonic patients. Caregiver education on hygiene and wear, along with strong coordination between pediatric dentistry, neurology, and intensive care teams, is essential for optimal outcomes.

This case series has certain limitations that should be considered while interpreting the findings. The report included only three patients with different neurological conditions and varying appliance designs, which limits the ability to generalize the outcomes. Although clinical healing and reduction in self-inflicted trauma were observed, the outcomes were assessed mainly through clinical observation rather than standardized objective measures. Parameters such as lesion severity, appliance retention, patient comfort, feeding improvement, and caregiver satisfaction were not quantitatively evaluated. In addition, the follow-up period was relatively short. Therefore, further studies involving larger sample sizes, longer follow-up periods, and standardized outcome assessment tools are needed to better understand the long-term effectiveness and clinical applicability of customized intraoral appliances in children with neurological disorders.

## Conclusions

Children with FIRES, GA-I, and post-neurosurgical involuntary movements exhibit a higher risk of self-inflicted oral trauma. Customized intraoral appliances - particularly those fabricated using digital technologies - offer an effective, conservative approach for tissue protection and trauma prevention. The cases presented demonstrate positive healing outcomes, excellent tolerance, and sustained prevention of recurrence. However, these findings are based on a limited number of cases with relatively short follow-up durations. Therefore, larger studies with longer follow-up periods and standardized objective outcome measures are required to further evaluate the long-term effectiveness, safety, and clinical applicability of these customized appliances in pediatric patients with neurological disorders.
